# Sex-specific differences in the genetic and environmental effects on cardiac phenotypic variation assessed by echocardiography

**DOI:** 10.1038/s41598-023-32577-6

**Published:** 2023-04-08

**Authors:** Honghuang Lin, Alan C. Kwan, Cecilia Castro-Diehl, Meghan I. Short, Vanessa Xanthakis, Ibrahim M. Yola, Gerran Salto, Gary F. Mitchell, Martin G. Larson, Ramachandran S. Vasan, Susan Cheng

**Affiliations:** 1 Department of Medicine, University of Massachusetts Chan Medical School, Worcester, MA USA; 2 Framingham Heart Study, Framingham, MA USA; 3 Department of Cardiology, Cedars-Sinai Medical Center, 127 S. San Vicente Blvd, Suite A3100, Los Angeles, CA 90048 USA; 4 Section of Preventive Medicine and Epidemiology, Department of Medicine, Boston University School of Medicine, Boston, MA USA; 5 Glenn Biggs Institute for Alzheimer’s & Neurodegenerative Diseases, University of Texas Health San Antonio, San Antonio, TX USA; 6 Department of Biostatistics, Boston University School of Public Heath, Boston, MA USA; 7 Cardiovascular Engineering, Inc., Norwood, MA USA; 8 Section of Cardiovascular Medicine, Department of Medicine, Boston University School of Medicine, Boston, MA USA; 9 Department of Epidemiology, Boston University School of Public Heath, Boston, MA USA; 10 Center for Computing and Data Sciences, Boston University, Boston, MA USA

**Keywords:** Cardiovascular genetics, Cardiology, Heart failure

## Abstract

The drivers of sexual dimorphism in heart failure phenotypes are currently poorly understood. Divergent phenotypes may result from differences in heritability and genetic versus environmental influences on the interplay of cardiac structure and function. To assess sex-specific heritability and genetic versus environmental contributions to variation and inter-relations between echocardiography traits in a large community-based cohort. We studied Framingham Heart Study participants of Offspring Cohort examination 8 (2005–2008) and Third Generation Cohort examination 1 (2002–2005). Five cardiac traits and six functional traits were measured using standardized echocardiography. Sequential Oligogenic Linkage Analysis Routines (SOLAR) software was used to perform singular and bivariate quantitative trait linkage analysis. In our study of 5674 participants (age 49 ± 15 years; 54% women), heritability for all traits was significant for both men and women. There were no significant differences in traits *between* men and women. Within inter-trait correlations, there were two genetic, and four environmental trait pairs with sex-based differences. Within both significant genetic trait pairs, men had a positive relation, and women had no significant relation. We observed significant sex-based differences in inter-trait genetic and environmental correlations between cardiac structure and function. These findings highlight potential pathways of sex-based divergent heart failure phenotypes.

## Introduction

Clinical manifestations and presentations of heart failure (HF) are known to differ between women and men, with women more frequently developing HF with preserved ejection fraction (HFpEF)^1^. Over the lifespan, women compared to men are found to have higher degrees of left ventricular (LV) thickening and stiffening as well as higher levels of circumferential shortening and torsion with aging^2–4^. These alterations manifest as measurable differences in LV structure and function between men and women, with greater concentric remodeling and diastolic dysfunction seen in women^5^. The underlying determinants of these sex differences remain incompletely understood. Accumulating evidence points to the likely role of sexual dimorphism in gene expression as well as response to environmental factors^6,7^. To this end, quantitative linkage trait analysis can be used to assess heritability as well as relative contributions of shared genetic and environmental influences to quantitative measures of cardiac phenotypes and sex differences in this regard, assuming the availability of pedigree information^8,9^. Therefore, the objective of this study was to investigate sex-specific differences in heritability as well as genetic versus environmental contributions to variation in cardiac structural and functional traits measured by echocardiography. By identifying these sex differences in a large community-based cohort, we aim to further understand the potential underlying drivers of differences that can result in divergent HF phenotypes consistently seen in clinical practice.

## Results

The study population included 5674 participants (mean age 49 ± 15 years; 54% women). As previously seen in sex-pooled analyses, heritability tended to be higher for cardiac structural traits than for cardiac functional traits^10^. For men, heritability ranged from 0.33 (for left ventricular wall thickness [LVWT]) to 0.57 (aortic root diameter [AoD]) for cardiac structural traits, and from 0.17 (mitral annular plane systolic excursion [MAPSE]) to 0.44 (log longitudinal synchrony [LSS]) for ventricular functional measures (*P* < 0.0001 for all) (Table 1). Heritability estimates for women appeared somewhat lower but after accounting for multiple testing were statistically similar for structural traits, ranging 0.26 (LVWT) to 0.39 (LV diastolic dimension [LVDD]), as well as for functional traits, ranging 0.22 (global longitudinal strain [GLS]) to 0.48 (log[LSS]). However, heritability of one particular structural trait, AoD, was significantly higher in men compared to women (0.57 ± 0.05 vs 0.37 ± 0.05, *P* = 0.01).

**Table 1:** Sex-specific heritability estimates.

Echocardiographic trait	Men		Women		*P value* (sex difference)
	Heritability estimate (h2)	*P value*	Heritability estimate (h2)	*P value*	
Cardiac structural traits					
Log(LVMi)	0.35 ± 0.05	1.7 × 10^−14^	0.36 ± 0.05	3.2 × 10^−16^	0.93
LVDD	0.34 ± 0.05	2.6 × 10^−12^	0.39 ± 0.05	3.0 × 10^−17^	0.55
LVWT	0.33 ± 0.05	8.6 × 10^−12^	0.26 ± 0.05	7.3 × 10^−10^	0.36
LAD	0.41 ± 0.05	2.4 × 10^−16^	0.34 ± 0.05	4.4 × 10^−14^	0.33
AoD*	0.57 ± 0.05	5.3 × 10^−29^	0.37 ± 0.05	1.7 × 10^−16^	0.01
LV functional traits					
LVEF	0.22 ± 0.06	2.6 × 10^−5^	0.25 ± 0.05	3.9 × 10^−9^	0.71
GCS	0.30 ± 0.05	1.2 × 10^−9^	0.24 ± 0.05	3.8 × 10^−8^	0.42
GLS	0.24 ± 0.05	1.2 × 10^−6^	0.22 ± 0.05	3.0 × 10^−7^	0.78
Log(LSS)	0.44 ± 0.05	2.6 × 10^−18^	0.48 ± 0.05	6.3 × 10^−28^	0.59
MAPSE	0.17 ± 0.05	3.7 × 10^−4^	0.27 ± 0.05	1.7 × 10^−9^	0.22
Log(E/e′)	0.30 ± 0.06	7.4 × 10^−9^	0.30 ± 0.05	1.5 × 10^−11^	0.93

*Heritability of AoD is higher in men compared to women (*P* = 0.01).

We also examined genetic and environmental correlations between trait pairs separately by sex. As shown in Table 2, strong but expected genetic correlations were observed for several pairs or cardiac traits. In men, genetic correlations were seen for certain pairs of cardiac function traits: − 0.89 ± 0.11 (global circumferential strain [GCS] with LV ejection fraction [LVEF]), and structural traits: 0.76 ± 0.06 (LVWT with LVMi) and 0.72 ± 0.06 (LVDD with LV mass index [LVMi]); these pairs were expected due to the inherent correlations between these measurement methods. In women, the strongest genetic correlations between cardiac traits were similar: − 0.71 ± 0.10 (GCS with LVEF), 0.67 ± 0.06 (LVWT with LVMi), and 0.81 ± 0.05 (LVDD with LVMi). For environmental correlations, significant and strongly associated findings were also noted in expected pairs: 0.64 ± 0.03 (LVWT with LVMi) and − 0.28 ± 0.05 (GCS with LVEF) for men with similarly significant correlations of 0.68 ± 0.03 (LVWT with LVMi) and − 0.37 ± 0.04 (GCS with LVEF) for women.

**Table 2:**
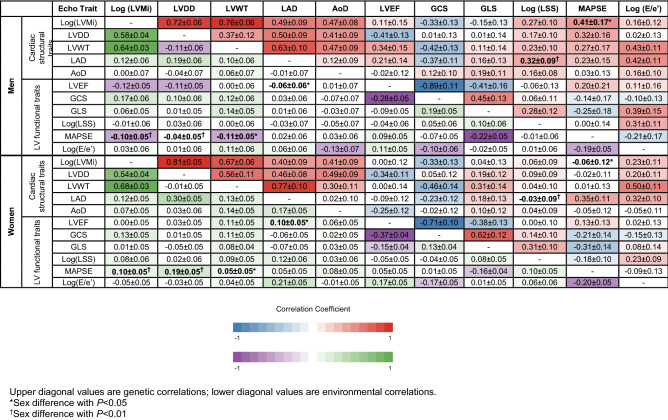
Sex-specific genetic/environmental correlations among echocardiographic traits.

Upper diagonal values are genetic correlations; lower diagonal values are environmental correlations.

*Sex difference with *P* < 0.05.

^†^Sex difference with *P* < 0.01.

**Figure 1:**
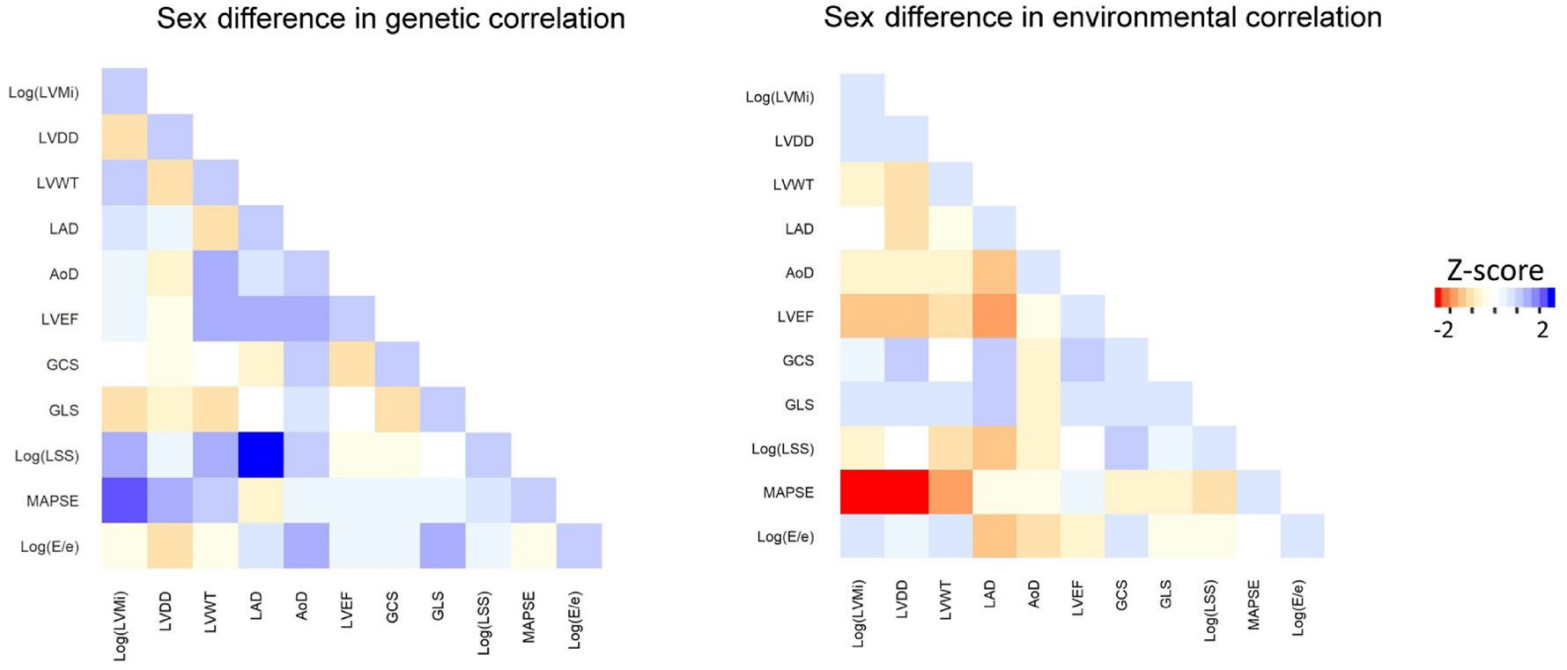
Sex differences in the genetic and environmental correlations among echocardiographic traits. Correlation values are represented on a color scale where blue represents higher correlation for men and red represents higher correlation for women.

Sex differences were seen for two genetically influenced and four environmentally influenced cardiac trait pairs (Fig. 1), after accounting for multiple testing. For both of the genetic trait pairs that demonstrated sex differences, men had a positive relation, and women had no significant relation: left atrial systolic dimension [LAD] with log[LSS] (men: 0.32 ± 0.09, women: − 0.03 ± 0.09, *P*_*difference*_ = 0.006), and MAPSE with LVMi (men: 0.41 ± 0.17, women: − 0.06 ± 0.12, *P*_*difference*_ = 0.04). For the four environment trait pairs that demonstrated sex differences, men had an inverse relation and women had no significant relation in one: LVWT with MAPSE (men: − 0.11 ± 0.05, women: 0.05 ± 0.05, *P*_*difference*_ = 0.03); men had no significant relation and women had a positive relation in two: LVDD with MAPSE (men: − 0.04 ± 0.05, women: 0.19 ± 0.05, *P*_*difference*_ = 0.002) and LAD with LVEF (men: − 0.06 ± 0.06, women: 0.10 ± 0.05, *P*_*difference*_ = 0.05); and, there was a significant sex-discordant relation observed for one trait pair: MAPSE with LVMi (men: − 0.10 ± 0.05, women: 0.10 ± 0.05, *P*_*difference*_ = 0.006).

## Discussion

The mechanistic underpinnings of sex-specific differences in HF phenotypes remains unclear. Our study, which represents a large-scale sex-specific analysis of heritability and genetic-environmental correlation of cardiac phenotypes, offers a few salient observations regarding differences in cardiac inter-trait correlations between men and women. First, men appeared to have slightly higher heritability than women for multiple cardiac traits, although these sex differences were not statistically significant after accounting for multiple testing. Sex differences in the genetic contribution to cardiac trait variation were more evident in inter-trait analyses; for genetic correlations, significant MAPSE-log(LVMI) and log(LSS)-LAD associations were seen in men, but not women. Second, all sex differences were observed for cardiac structure–function relations, not for cardiac structure–structure or function–function relations. Most associations involved specific measures of cardiac myocardial mechanical motion such as LV longitudinal shortening (LSS) and with four of the six differences involving MAPSE. Finally, and notably, there was one association that demonstrated a particularly significant and opposite sex difference, wherein the environmental correlation of LVMi and MAPSE was significantly positive for women and significantly negative for men.

Recognizing that we are unable to infer the directionality of the observed cardiac structure–function associations, our results are both illuminating and hypothesis generating regarding the potential timing and pathways by which sex-specific HF phenotypes arise. The findings that inter-related structure–function measures could be more genetically influenced in men than women suggests that if men develop relations between structure and function resulting in subsequent HF, they may do so at a younger age (i.e., less influenced by cumulative risk exposures including environmental factors over the life course). In turn, the inter-relations of LV structural and functional traits appearing more environmentally influenced in women suggests that if women are likely to develop integrated LV structural and functional abnormalities and subsequent HF, these may occur after longer duration of cumulative risk exposures including environmental factors over the life course. Interestingly, there is concordant evidence for female compared to male vascular physiology being potentially more susceptible to cardiometabolic risk burden over the adult life course^11^. In the current analysis, the finding that women and men had actually directionally opposite as well as significant findings regarding the environmentally mediated correlations between LVMi and MAPSE underscores how environment influences on both sexes likely drive the classic pathological remodeling patterns seen in men versus women—with women showing more concentric remodeling and men with eccentric remodeling. Increases in LVMi associated with increased longitudinal movement are more consistent with a concentric pathway, as commonly seen in aging women, whereas increases in LVMi associated with decreased longitudinal movement are more consistent with an eccentric pathway, as more often seen in men. Although apparently paradoxical, the inverse LVMi and MAPSE association in women is concordant with the LVDD and MAPSE association in women—given that the most commonly seen pattern of age-related concentric remodeling in women typically involves decreasing LVDD (and, in turn, LVMi) in association with decreasing MAPSE. Notwithstanding the subtleties required to interpret these results, it is notable that each of the sex-divergent patterns appears responsive to environmental effects, particularly the more clinically adverse form of eccentric remodeling in men. Overall, these findings highlight the continued importance of assessing measures of longitudinal ventricular motion in the context of all aspects that govern longitudinal motion (e.g. relative fiber orientation and synchrony) when investigating sex differences in HF phenotypes. These findings also underscore the potential for further discovery of the mediators and pathways by which observed sex differences develop over time into divergent subclinical HF phenotypes as well as divergent clinical HF outcomes.

Our study expands from the previously established body of evidence demonstrating heritability of echocardiographic traits. In particular, our results underscore the need for sex-specific analyses in future genome-wide association studies to further interrogate sex differences in cardiac structure and function that are consistently observed in clinical practice. Intriguingly, our finding of sex-specific heritability of aortic root size coincides with recently reported sex-specific polygenic risks score associations with hypertension traits in a separate cohort^12^. Taken together, these emerging data suggest that sex differences in vascular as well as associated cardiac traits and outcomes may have discernible sex-specific genetic underpinnings that could eventually, pending future follow-up work, serve as a premise for considering sex-specific approaches to cardiovascular risk assessment and management.

There are limitations of our study that are worth noting. Our analyses focused on sex differences in overall genetic contribution and in the overall environmental contribution to cardiac traits; thus, future follow-up work is also needed to examine the contributions of specific individual genetic variants and individual environmental factors underlying our findings. While our analyses focused on established left heart measures, it is now recognized that left- and right-heart cardiac alterations tend to be inter-related given their physiologic as well as anatomic inter-connectedness within the closed pericardial space. Historically fewer echocardiography studies have focused on right heart measures, and so additional investigations are needed to determine whether similar or different sex-specific genetic or environmental effects may be seen in relation to right heart traits. Importantly, given that the participants of our study are predominantly white individuals of European ancestry, replication work in more diverse study samples are needed to validate our findings. We also recognize that the sensitivity of identifying additional sex-specific associations may have been limited by our study sample size. Therefore, additional follow-up studies in large-sized samples are needed to not only validate but also potentially expand our findings. Although heritability estimates are challenging to validate outside of studies that have multi-generational family structure similar to that of the Framingham Heart Study, our results suggest that follow-up genome wide association studies in separate community-based cohorts would be useful for confirming that sex-specific associations with cardiac traits exist for individual or groups of genetic variations.

In summary, we found similar overall heritability between men and women with respect to cardiac function and structural traits, with some notable sex differences in cardiac inter-trait correlations. In particular, men tended to demonstrate more genetically linked associations between cardiac structure and function, and women tended to demonstrate more environmentally influenced cardiac structural–functional associations. These findings from a single limited-sized cohort study need to be replicated, expanded, and further investigated in larger-sized and more diverse cohorts. Nonetheless, the results are intriguing and underscore the potential to identify potentially discordant gene-environment determinants that may be contributing to the sex differences in subclinical and clinical cardiac disease conditions and outcomes that are consistently observed in population studies and that are commonly encountered among individual patients in practice.

## Methods

We studied the sex-specific relations of cardiac structure and function within the Framingham Heart Study (FHS) participants of Offspring Cohort examination 8 (2005–2008) and Third Generation Cohort examination 1 (2002–2005). We measured cardiac traits using standardized echocardiography with measurements performed in the FHS laboratory according to American Society of Echocardiography guidelines^13^. As in prior genetic analyses of echocardiographic traits^14^, we excluded individuals with prevalent HF (n = 71) or any missing echocardiographic measure or covariates (n = 1351). Given that individuals with prevalent HF can have extreme forms of cardiac remodeling and dysfunction related to dynamic disease processes or their treatments^14^, we excluded individuals with prevalent HF identified prior to or at the time of echocardiography. Prevalent HF was defined according to published criteria including signs and symptoms or a documented history of HF^15^. For our linkage analyses, we included five cardiac structural traits (left ventricular mass index [LVMi], LV diastolic dimension [LVDD], LV wall thickness [LVWT], left atrial [LA] systolic dimension, and aortic root diameter [AoD])^16^ and six functional traits (LV ejection fraction [LVEF], global circumferential and longitudinal strain [GCS, GLS], longitudinal synchrony [LSS], mitral annular plane systolic excursion [MAPSE], and mitral E/e’ ratio). LVWT was calculated as the sum of septal wall thickness and posterior wall thickness, consistent with prior similar genetic analyses of echocardiographic traits^14^”.

We used the Sequential Oligogenic Linkage Analysis Routines (SOLAR) program to perform the singular and bivariate quantitative trait linkage analysis. SOLAR is a flexible software which uses familial structures from pedigrees to perform linkage analysis between quantitative traits. It can account for multiple loci, dominance and household effects, and interactions^8^. For the current analyses, the input data into SOLAR required two files. The first file was the pedigree file, which listed the pedigree structure of study participants, which includes information on parents and family as detailed previously^17^. The second file was the phenotype file, which included information on the phenotype to be studied. The genetic heritability was estimated using the SOLAR software “polygen” function; the genetic and environmental correlation between any two traits was estimated using the “polygen–testrhoe–testrhog” function. For each echocardiographic trait, a linear regression model was used to adjust for the effect of age, sex and height. The residuals were then transformed by the rank-based inverse normal transformation. Due to the skewed distributions of LVMi, LSS and E/e′, these traits were natural log-transformed before the linear regression adjustment. We used the variance components method to estimate the genetic and environmental correlations between echocardiographic traits^10^. Sex-differences were assessed by t-test, and Bonferroni correction was used to adjust for multiple testing.

## Data Availability

The data used in the current study could be requested through the dbGaP database with Study Accession: phs000007.v32.p13.

## Abbreviations

AoD: Aortic root diameter
GCS: Global circumferential strain
GLS: Global longitudinal strain
LAD: Left atrial systolic dimension
LSS: Longitudinal synchrony
LV: Left ventricle/left ventricular
LVDD: Left ventricular diastolic dimension
LVEF: Left ventricular ejection fraction
LVM: Left ventricular mass
LVMi: Left ventricular mass index
LVWT: Left ventricular wall thickness
MAPSE: Mitral annular plane systolic excursion
SOLAR: Sequential Oligogenic Linkage Analysis Routines
